# Washing COVID-19 away: COVID-19 and obsessive compulsive disorder

**DOI:** 10.1192/j.eurpsy.2021.776

**Published:** 2021-08-13

**Authors:** R. André, M.J. Gonçalves, C. Sereijo, M. Abreu

**Affiliations:** 1 Psychiatry, Centro Hospital Lisboa Norte, Lisboa, Portugal; 2 Psiquiatria, Centro Hospitalar Universitario Lisboa Norte, oeiras, Portugal; 3 Psiquiatria, Centro Hospitalar Universitario Lisboa Norte, Lisboa, Portugal

**Keywords:** obsessive compulsive disorder, ocd, COVID19, pandemic

## Abstract

**Introduction:**

We are facing a crisis caused by an extremely infectious disease, Covid-19. The mechanisms of infection and transmission of this coronavirus are largely unknown but some of the clearer recommendations are washing hands and surfaces. Obsessive-Compulsive Disorder has a lifetime prevalence of 2-3%, among the multiple symptoms, fear of dirt or being contaminated, and excessive washing are the most common affecting about 50% of patients.

**Objectives:**

We reviewed the available information to understand if there are changes in OCD symptoms during the pandemic.

**Methods:**

Non-systematic review of the literature with selection of scientific articles published in the past 6 months; by searching Pubmed and Medscape databases using the combination of MeSH descriptors. The following MeSH terms were used: Covid-19; SARS-Cov2; pandemic; obsessive compulsive disorder; OCD.

**Results:**

From a theoretical point of view, the increased frequency of hand washing and the importance of following hand-washing steps can add to a ritualistic pattern, also cleaning hands every time a person comes from outside or contacts with others can be justified as a preventive action rather than considered a problem and it can be “normalized” by others as a pandemic response.

**Conclusions:**

In conclusion, there is evidence that during quarantine an overall increase in obsession and compulsion severity emerged with contamination symptoms associated with worse outcomes. There is data on an increase in relapses with patients not asking for help in a timely manner. The current situation is unpredictable and rapidly changing. It is likely that more information about this topic will arise in the next months.

